# Implementation of remote home care: assessment guided by the RE-AIM framework

**DOI:** 10.1186/s12913-024-10625-9

**Published:** 2024-01-29

**Authors:** Lina Oelschlägel, Anne Moen, Alfhild Dihle, Vivi L. Christensen, Kristin Heggdal, Jane Österlind, Simen A. Steindal

**Affiliations:** 1 Lovisenberg Diaconal University College, Lovisenberggata 15B, Oslo, 0456 Norway; 2 Department of Public Health Sciences, Institute of Health and Society, Faculty of Medicine, University of Oslo, Oslo, Norway; 3 Department of Nursing and Health Promotion, Faculty of Health Sciences, OsloMet - Oslo Metropolitan University, Oslo, Norway; 4 Department of Nursing and Health Sciences, Faculty of Health and Social Sciences, University of South-Eastern Norway, Drammen, Norway; 5 Faculty of Health Sciences, VID Specialized University, Oslo, Norway; 6 Department of Healthcare Sciences/Palliative Research Center, Marie Cederschiöld University, Stockholm, Sweden

**Keywords:** Assessment, Health care technology, Home-based, Palliative care, Qualitative, Reach effectiveness adoption implementation maintenance (RE-AIM) framework

## Abstract

**Background:**

Welfare technology interventions have become increasingly important in home-based palliative care for facilitating safe, time-efficient, and cost-effective methods to support patients living independently. However, studies evaluating the implementation of welfare technology innovations are scarce, and the empirical evidence for sustainable models using technology in home-based palliative care remains low. This study aimed to report on the use of the Reach Effectiveness Adoption Implementation Maintenance (RE-AIM) framework to assess the implementation of remote home care (RHC) a technology-mediated service for home-living patients in the palliative phase of cancer. Furthermore, it aimed to explore areas of particular importance determining the sustainability of technologies for remote palliative home-based care.

**Methods:**

A secondary analysis of data collected by semi-structured interviews with patients with cancer in the palliative phase, focus groups, and semi-structured interviews with healthcare professionals (HCPs) experienced with RHC was performed. A deductive reflexive thematic analysis using RE-AIM dimensions was conducted.

**Results:**

Five themes illustrating the five RE-AIM dimensions were identified: (1) Reach: protective actions in recruitment - gatekeeping, (2) Effectiveness: potential to offer person-centered care, (3) Adoption: balancing high touch with high tech, (4) Implementation: moving towards a common understanding, and (5) Maintenance: adjusting to what really matters. The RE-AIM framework highlighted that RHC implementation for patients in the palliative phase of cancer was influenced by HCP gatekeeping behavior, concerns regarding abandoning palliative care as a high-touch specialty, and a lack of competence in palliative care. Although RHC facilitated improved routines in patients’ daily lives, it was perceived as a static service unable to keep pace with disease progression.

**Conclusions:**

A person-centered approach that prioritizes individual needs and preferences is necessary for providing optimal care. Although technologies such as RHC are not a panacea, they can be integrated as support for increasingly strained health services.

## Background

Palliative care adopts a holistic perspective, aiming to maintain quality of life for people living with severe illnesses that limit life expectancy, such as cancer, by relieving physical, psychosocial, spiritual, and existential suffering [1]. Palliative care has evolved from focusing on the care of the dying, to emphasizing early integration of palliative care for patients with cancer [2]. Palliative care should be based on patients’ personal preferences, which requires a person-centered approach from healthcare professionals (HCPs) [3, 4]. As most patients who need palliative care prefer to remain at home for as long as possible, current health strategies for implementing palliative care within primary care aim to support these patients and their preference to live and receive long-term treatment at home [5].

To meet challenges in an increasingly strained health system, where higher workloads and fewer healthcare professionals, establishing innovative solutions for providing palliative care has become essential [6, 7]. Many terms can be used to reference technological innovations. In this study, we use the term welfare technology. Welfare technology is an umbrella term, mainly used in Nordic countries [8–10] and refers to technologies interacting with all stakeholders (including patients, families/informal caregivers and HCPs) involved in the care service; not only to support care, but also to alter how care is provided [11]. Remote home care (RHC) is an example of an innovative welfare technology as a supportive service for home-living patients with cancer in the palliative with the intention of enabling patients to stay in their homes for as long as possible. The term RHC is a direct translation of a commonly used Norwegian term for welfare technology supported homecare. “Remote” conveys that HCPs are situated in different geographic locations from that of the patient, whereas “home care” refers to the service being available in the patient’s own home. RHC provides tailored follow-ups and improves communication between patients and HCPs. RHC is a non-ambulant service based on three components: (1) a tablet device containing individualized questions for self-reporting symptoms, (2) sensor data via medical measuring devices (such as weight scales, pulse oximeters, blood glucose meters, blood pressure monitors, and electronic drug dispensers), and (3) patient-HCP communication via chat or telephone.

Although empirical evidence for digital interventions beneficial to home-based palliative care is growing [12, 13], the adoption of technological innovations in healthcare has been slower than expected in most countries [14]. Moreover, there has been limited prioritization and publication of studies on innovative interventions such as the RHC, indicating a significant knowledge gap regarding the potential of technology to enhance sustainable patient outcomes in palliative care [15, 16]. Without the right evaluation tools, there is a chance of welfare technology being implemented as an end in itself, rather than as a method for improved care [17]. Thus, research that integrates the effectiveness of interventions with ways to successfully incorporate them into existing organizational contexts is necessary. This type of research is known by various names in the literature, such as implementation science, dissemination science, translational research, and knowledge transfer [18]. The Reach Effectiveness Adoption Implementation Maintenance (RE-AIM) [19] framework is useful in this type of research.

### RE-AIM framework

The RE-AIM framework [19] is an acronym of five evaluative dimensions that describe the overall population-based impact of an intervention such as RHC; the individual level (those who the intervention is intended to benefit), and the staff and setting levels (the institution applying the intervention) [20, 21]. The RE-AIM was developed and deployed to assist in the planning, management, evaluation, and reporting of studies supporting the translation of research or innovations into practice [18]. The framework seeks to balance the traditional focus of internal over external validity and facilitate sustainable adoption and implementation of effective, generalizable, and evidence-based interventions [21]. Table 1 illustrates the dimensions, levels, and definitions of the RE-AIM framework.

**Table 1:** RE-AIM dimensions, levels, and definitions

RE-AIM dimension and level	Definition
**Reach** Individual	Representativeness, rate, and characteristics of individuals who are willing to participate in a given intervention, including potential barriers for participation
**Effectiveness** Individual	Impact of an intervention on individual outcomes, such as positive and negative effects, quality of life, and economic outcomes
**Adoption** Institutional	Representativeness and proportion of settings that implement the intervention
**Implementation** Institutional	Institutional fidelity to intervention protocols, consistency in intervention delivery, and timing and cost of the intervention
**Maintenance** Individual + institutional	Extent to which the intervention has become institutionalized or part of routine organizational practices and policies. In addition, individuals address the long-term effects of the intervention outcomes following intervention completion

RE-AIM: Reach, Effectiveness, Adoption, Implementation and Maintenance [21]

Although the RE-AIM framework has been widely employed for planning, managing, and evaluating a large number of interventions in the past two decades [21], the published literature reveals a shortage of qualitative approaches using RE-AIM [22–24]. The RE-AIM dimensions highlight the need to measure not only traditional clinical outcomes, such as effectiveness, but also implementation outcomes, which are crucial for ensuring widespread public health impact. Holtrop et al. [22] argues that qualitative approaches may help results and provide answers regarding why and how implementation processes unfolded the way they did. Thus, qualitative studies using the RE-AIM framework can provide a deeper insight into the intended and unintended outcomes of new welfare technologies, such as RHC, to support home-based palliative care. Such valuable information can contribute to the translation of relevant interventions into practice [22].

The aim of this study is to report on the use of the RE-AIM framework to assess the implementation of RHC, a technology-mediated service for home-living patients in the palliative phase of cancer. Additionally, the study explores areas of particular importance in determining the sustainability of technologies for remote palliative home-based care.

## Methods

This study was based on a secondary deductive reflexive thematic analysis [25] using the RE-AIM framework [19] to assess experiences with RHC implementation for patients in the palliative phase of cancer. The assessment was based on qualitative data obtained from individuals, focus group interviews with patients, and HCPs; and is part of a project exploring patients’ and HCPs’ experiences with using RHC in palliative homecare. Comprehensive explanations of RHC intervention and setting and participant recruitment details were published in two recent studies [4, 26]. The Consolidated Criteria for Reporting Qualitative Research (COREQ) checklist guided the reporting for this study [27].

### Setting and recruitment

The study sample was recruited from one homecare district in a municipality situated in eastern Norway. The RHC service team is an independent community care service offering only RHC and is not affiliated with traditional homecare services. The RHC service team is experienced with offering RHC to home-living patients with diverse chronic illnesses. However, RHC delivery to patients in the palliative phase of cancer commenced in September 2017, upon enrollment of the initial patient for this study. When included in the study, patients with cancer in the palliative phase received RHC as a supplement, not a replacement for, standard palliative healthcare services.

The participants were recruited through purposeful sampling [28]. Initially, there was an intention to include family members as study participants. However, only two agreed to participate, thus we decided not to include family members in the study. The sample consisted of 11 patients in the palliative phase of cancer and 8 interdisciplinary HCPs employed by the RHC service team [4, 26].

RHC devices were provided according to each patient’s perceived needs and therefore varied. Before implementing RHC for patients in the palliative phase of cancer, most HCPs had prior experience with RHC for patients with various chronic illnesses. However, their familiarity with palliative care and cancer patients was comparatively limited. While a few HCPs possessed more expertise in cancer and palliative care, they were relatively less experienced with RHC. Thus, at the time of data collection, all the included HCPs were familiar with different aspects of RHC service for patients in the palliative phase of cancer. The sample characteristics are listed in Table 2. The authors had no relationships with the participants prior to their inclusion in this study.

**Table 2:** Characteristics of the patients in the palliative phase of cancer and interdisciplinary HCPs (*N* = 19)

Patients characteristics (*n* = 11)		HCP characteristics (*n* = 8)	
**Sex**		**Sex**	
Females	5	Females	6
Males	6	Males	2
**Age, years**		**Age, years**	
Mean (range)	66 (30–94)	Mean (range)	37 (27–50)
**Living-situation**		**Profession**	
Cohabiting	4	Specialized nurse^a^	2
Living alone	7	Nurse	2
**RHC devices provided**		Social worker	1
Tablet device	2	Physical therapist	1
Tablet device with self-reporting of symptoms	9	Occupational therapist^b^	2
Weight scale	6	**Experience in healthcare**	
Electronic drug dispenser	2	Years, mean (range)	13 (4–27)
Blood glucose meter	1	**Experience in current position**	
Pulse oximetry	1	Years, mean (range)	6 (1–10)
Blood pressure monitor	1		

^a^ One specialized nurse operated as cancer care coordinator in the district

^b^ One occupational therapist operated as project manager for implementing RHC.

HCP, healthcare professional; RHC, remote home care

### Data collection

The senior author (SAS) collected patient data by conducting 35 repeated individual interviews over the 16-week intervention period between September 2017 and March 2019. The first interview with each participant took place shortly after the RHC home follow-up was established (T1), followed by interviews at weeks 4 (T2), 12 (T3), and 16 (T4) of RHC use. Five patients were interviewed on all occasions; one patient only participated at baseline. Table 3 presents an overview of the patient interviews.

**Table 3:** Overview of patients participating in interviews

Patient	Interviewed at baseline (T1)	Interviewed at 4 weeks (T2)	Interviewed at 12 weeks (T3)	Interviewed at 16 weeks (T4)
Patient 1	X	X	X	X
Patient 2	X	X	X	X
Patient 3	X	X	X	X
Patient 4	X	X	X	
Patient 5	X	X	X	X
Patient 6	X	X		X
Patient 7	X	X	X	X
Patient 8	X	X		
Patient 9	X	X	X	X
Patient 10	X			
Patient 11	X	X		
Total number of patients per interview	*n* = 11	*n* = 10	*n* = 7	*n* = 7

HCP data was collected retrospectively. The first author (LO) collected data from the HCPs through focus groups and individual interviews in November 2019. Important topics identified from audio recordings and transcribed material were used to facilitate individual interviews with six of the eight focus group participants. All the interviews used a semi-structured interview guide consisting of open-ended and probing questions covering topics related to participant experiences with RHC. The interviews were audio recorded, transcribed verbatim, and checked for accuracy by the authors (LO and SAS). Interview guidelines are available upon request from the first author (LO).

### Ethics

The study was approved by the Norwegian Centre for Research Data (NSD) (reference number: 429,408) and exempted from review by the Norwegian Regional Committee for Medical and Health Research Ethics (REK). All the participants received oral and written information assuring confidential and voluntary study participation with the opportunity to withdraw from the study at any time. Details of the participants were kept separate and locked. Data were managed and stored securely, following the guidelines set forth by the NSD. Taking anonymization and confidentiality into consideration, this paper mainly refers to the HCP group as a whole: the RHC service team.

### Analyses

The data from the patients and HCPs used in this study were previously analyzed separately by a qualitative content analysis [29]. Results from these analyses are published in two separate publications that focus on patient and HCP experiences using RHC in palliative homecare [4, 26]. To assess RHC implementation specifically for supporting palliative care and to explore areas of particular importance for sustainability of welfare technologies for palliative homecare care, the transcribed data material from the patients and HCPs were imported to NVivo 12 for a secondary analysis. A deductive reflexive thematic analysis [25], using the RE-AIM framework [19], was applied. The RE-AIM dimensions Reach, Effectiveness, Adoption, Implementation, and Maintenance (Table 1) guided the analysis of the following six phases:

#### Phase 1: dataset familiarization

A deductive approach was initiated by applying the five RE-AIM dimensions to the existing patient and HCP datasets. This provided an initial structure, in which the data extracted from the two datasets were assessed and placed within a given RE-AIM dimension. The data extracts were read, re-read, and re-arranged multiple times to determine meanings and patterns across the datasets.

#### Phase 2: generating codes

To capture the meanings in the dataset, the initially structured data extracted from the patients and HCPs were coded using semantic codes. The semantic codes were revised and processed before being labelled with latent codes to capture their implicit meanings [25]. The latent codes were revised and re-arranged according to the five RE-AIM dimensions.

#### Phases 3 and 4: constructing and reviewing potential themes

**Fig. 1:**
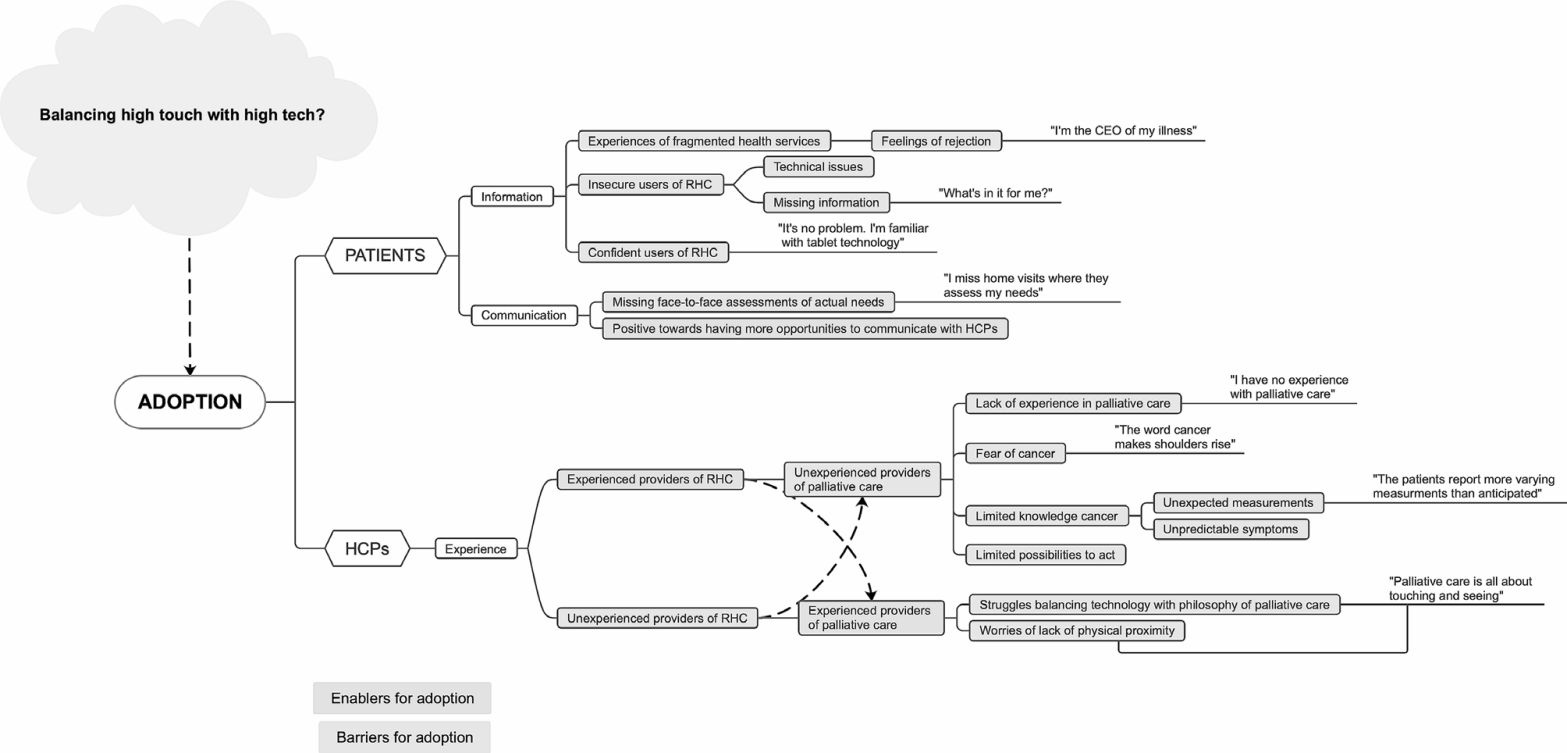
Example of visual mapping exploring themes related to adoption

Using the qualitative RE-AIM data questions suggested by Holtrop et al. [22] (Table 4), the latent codes were constructed and revised multiple times before collating into potential and later themes. Visual mapping was employed to provide an overview and explore the association of the potential themes with each other [25] (Fig. 1).

**Table 4:** Qualitative RE-AIM questions used in the analysis phases 3 and 4

RE-AIM Dimensions	Questions guiding the analysis
Reach	What factors contribute to the participation/non-participation of the participants? What might have been done to get more of the target audience to participate?
Effectiveness	Did the intervention work to effect the outcomes noted? What other factors contributed to the results? Are the outcomes found accurate? Are the results meaningful?
Adoption	What factors contributed to the organization and its individuals taking up the intervention? What barriers interacted with the intervention to prevent adoption? Was there partial or complete adoption? Why did some staff members in these organizations participate and others did not?
Implementation	How was the intervention implemented? By whom and when? What influenced implementation or lack of implementation? What combination of implementation effects affected the outcome results? How and why was the program or policy adapted or modified over time?
Maintenance	Is the intervention being implemented (and adapted) after the intervention core period? What is sustained, what discontinued, what modified- and why?

RE-AIM: Reach, Effectiveness, Adoption, Implementation, and Maintenance

Holtrop and Rabin [22]

#### Phase 5: defining and naming themes

The candidate themes were refined and revised in accordance with the RE-AIM dimensions to ensure that they highlighted important patterns across the dataset. This step, as well as the final step of defining and naming the themes, was performed collaboratively by a group of researchers (LO, AM, SAS). LO has work experience from neonatal intensive care, AM has research experience in welfare technology and service development, and SAS has clinical and research experience in cancer care and palliative care. None of these researchers have clinical experience with RHC in homecare use. The diverse research experiences enhanced and strengthened our analytic work regarding interpretations, analytic abstractions, intersubjectivity, and the credibility and dependability of the results [28]. The final analysis and interpretation were discussed in its entirety.

#### Phase 6: text preparation

The first author (LO) prepared the text presenting the preliminary results, which was thoroughly read, discussed, and subsequently revised in collaboration with all the authors. NVivo facilitated data storage and organization.

The sample size was determined using the theoretical model of information power suggested by Malterud et al. [30]. Information concerning the sample size of the relevant datasets are available in two recently published studies [4, 26].

## Results

The results for the secondary analysis of the data from the patients and HCPs are presented according to the five RE-AIM dimensions as follows:

1. Reach: protective actions in recruitment - gatekeeping.
2. Effectiveness: potential to offer person-centered care.
3. Adoption: balancing high touch with high tech.
4. Implementation: moving towards a common understanding.
5. Maintenance: adjusting to what really matters.

### Reach: protective actions in recruitment - gatekeeping

Reach considers factors contributing to participant participation/non-participation in the recruitment of individuals. Considering RHC, the Reach dimension applied to patients and HCPs who met the inclusion criteria, agreed to participate in the study, and received RHC as a supplement to standard palliative healthcare services.

Patients asked to participate were positive and found it meaningful to test a service that could potentially benefit others.

“I had little expectations. I thought that this [RHC and research participation] was something I could do for you. To the benefit of others later.”

Patient 10_ T1

HCPs in the RHC service team who were experienced using RHC for patients with chronic obstructive lung disease (COPD) and diabetes, regarded RHC as an opportunity to get closer to patients with cancer in the palliative phase living at home. However, some HCPs less experienced with RHC were skeptical of introducing high-tech services to care for these patients. This was evident in the recruitment of patients to RHC; HCPs less experienced with RHC felt a responsibility not to impose unnecessary stress and burden on patients, and therefore failed to introduce RHC to patients they perceived as very frail.

“I felt it was wrong to expose patients with cancer to this [RHC and research participation] in addition to everything else.”

HCP 4_ focus group 1

Furthermore, the HCPs involved in the recruitment expressed that it was challenging to anticipate and decide which patients could benefit from receiving RHC, and when in the palliative phase and disease trajectory it was appropriate to introduce RHC, even though the inclusion criteria were specified. These protective actions, or gatekeeping behaviors, in HCPs may have led to eligible patients missing the opportunity to receive RHC.

Over time, HCPs became more aware that their job was not so much to act but more to provide safety and stability to patients and pass on important information to others responsible for patient medical follow-up. HCPs experienced RHC as more beneficial to patients than initially anticipated and became more eager to introduce RHC to patients.

“In the beginning, we [the RHC service team] had a lot of resistance. Because palliative care is about meeting and seeing, you have to touch, feel, and interpret. You can’t do that from a distance. But now we see that it [RHC] becomes a comfort for the patient. There’s continuity, and they gain a certain level of control. Then there’s the aspect of mastery. They have control over their own lives. And they have the reassurance that they can reach out if there’s anything.”

HCP 5_focus group 2

### Effectiveness: RHC potential to offer person-centered care

The Effectiveness dimension considers any RHC impact on patient individual outcomes.

Patients stated that the RHC devices and statistics on the tablet computer provided an overview of symptom development, which improved their daily routines and contributed to enhanced feelings of security and reassurance that someone was paying attention to their situation. During the interviews at T1 and T2, most patients were satisfied with the training and information they received from the RHC service teams. However, during the RHC intervention period, several patients experienced changes in their conditions without the content of the RHC being updated or adjusted accordingly. This was considered discouraging, and patients became less inspired to submit their scores and measurements. Patients shared feelings of despair and uncertainty regarding their future. Some questioned how the RHC would benefit their situation if not adjusted to their altered conditions and preferences. At T4, several patients expressed confusion regarding RCH use.

“When I spoke to you and the project manager, I felt it was very difficult to understand what you really want with these questions and all that stuff.”

Patient 7_ T4

Most patients sought more opportunities to communicate face-to-face and expressed the need to communicate their needs in person, not merely by providing scores and statistics. Some patients expressed a desire to get to know another person on “the other side of the tablet computer.” Regarding face-to-face contact, the HCPs were divided; some felt that it was important to visit the patient physically, at least once, to assess the submitted data.

“I feel it’s easier to talk to them [patients] over the phone if I’ve seen them in person and been in their apartment and know what it looks like there. Then I can give advice based on that. I don’t know what it is, but it feels safer.

HCP 6_individual interview

Other HCPs felt it was possible to make assessments based on submitted data and telephone conversations.

“I feel like I know the patients very well, even though I haven’t met all of them. In telephone conversations I use their [patients] responses to tailor questions about symptoms and measurements from medical devices. This allows for a more genuine conversation compared to just asking about their week, as patients might recall only fragments. Having measurements makes conversations more meaningful and reliable.”

HCP 5_focus group 2

### Adoption: balancing high touch with high tech

The Adoption dimension considers the representativeness and willingness to initiate the intervention, which, in this context, refers to the perspectives of both the patients and HCPs who used RHC.

Patients expressed great self-confidence concerning RHC usability and related this to personal experiences with smartphone technology. Most patients seemed satisfied with the RHC user-friendliness, and the initial training and information provided by the RHC service team. However, there were barriers making full RHC Adoption challenging. Some patients experienced fragmented health service, especially if their health situation required the involvement of many health service staff. Some patients contacted the RHC service team with everyday problems, only to have then been told by the RHC service team to contact others. This fragmentation was experienced as problematic because patients did not seem to know how the RHC team defined their service and lacked knowledge of which service was responsible for what. For these patients, RHC represented yet another service that contributed to a corresponding increase in service complexity.

“I had feedback in relation to poor cleaning but was told that they [the RHC service team] couldn’t help me with it. I got a phone number, but I don’t know what’s going on. If there’s anyone who can help me at all.”

Patient 1_T3

Most HCPs were experienced RHC providers and had positive attitudes towards technology. Some stated that a positive attitude towards providing care through technology was essential for receiving an employment offer from the RHC service team. However, the inclusion of patients in the palliative phase of cancer added care requirements and led to changes in the HCP attitudes. The HCPs experienced with palliative care expressed skepticism concerning RHC introduction to these patients, especially because of the lack of physical proximity.

“Palliative care is about meeting and seeing. You have to touch and feel and interpret. It cannot be done remotely. It can create anxiety in the patient. My experience says you can’t do that.”

HCP 4_Focus group 1

Most HCPs were experienced in providing RHC to other patient groups; however, they had no prior training or experience with either palliative care or cancer care. They experienced that patients with cancer reported unexpected and fluctuating symptoms and measurements; moreover, HCPs described feelings of anxiety and an increased sense of responsibility.

“We didn’t have enough knowledge. I felt fearful when it came to this patient group. It just seems so much scarier when it’s about cancer. There are like 200 different diagnoses, and what they [the patients] report could be anything […] And as a healthcare professional, I feel a need to help.”

HCP 1_Individual interview

### Implementation: moving towards a common understanding

The Implementation dimension focuses on identifying facilitators and barriers related to ensuring consistency in RHC service delivery within the framework of the HCPs employed at the RHC service team.

The RHC was designed with a high level of flexibility, allowing for HCPs to tailor and adjust services according to each patient’s unique situation. The HCPs had prior experience utilizing RHC in the follow-up care of patients with COPD, where these individuals typically possessed a COPD Action Plan for self-treatment. This plan served as valuable support for HCPs in decision-making and assessments. However, the scenario differed for patients in the palliative phase of cancer. In this context, the absence of a standardized plan became evident, and addressing issues related to pain posed a particular challenge. Both patients and HCPs emphasized the necessity for tailored, branched questions that would enable patients to provide in-depth explanations, particularly regarding symptoms such as pain.

“If I have pain, I don’t get to elaborate on where the pain is when I tick the form. Shouldn’t that be somewhat important for the person who is going to assess the pain? Because otherwise they have to call me and find out where I’m in pain. I can probably describe the pain by sending an additional message.”

Patient 6_T1

Furthermore, HCPs learned that the sensor data, such as weight from a patient group whose weight is expected to decrease, provided little valuable information and questioned the purpose of collecting such data without the opportunity or mandate to act and take necessary action. These challenges became more prominent as the patient’s condition changed, and assessments had to be made. The HCPs often had no other option than to refer patients to the emergency room or general practitioners.

“If there’s a need for pain relief beyond the usual, we contact the patient’s doctor to notify.”

HCP 5_individual interview

HCPs constantly experienced challenges in gaining access to patient information, which resulted in much valuable time being spent on telephonic conversations attempting to obtain the necessary information. Several patients had general practitioners who had not heard of RHC, rendering collection of the necessary information, such as patient medication, challenging. Consequently, HCPs had to rely on the patients to provide them with information.

“We’re not notified of changes in treatment unless the patient tells us about it. We can recommend something they shouldn’t do anymore, so we pay close attention and time and ask the patient. It can be risky business.”

HCP 1_individiual interview

Our results indicated that RHC lacked anchoring in the healthcare service and that sufficient adaptations had not been made before patients with cancer in the palliative phase were included. HCPs expressed doubts about how they should act when patients reported unexpected measurements and limited possibilities for help. In situations where patient situations were perceived as unclear, several HCPs explained it could be difficult for them to leave work.

### Maintenance: adjusting to what really matters

Following the 16-week intervention study period, the homecare district under study continued RHC as a service for patients in the palliative phase of cancer. The HCP experiences resulted in implementing a more person-centered approach, with objective measurements given less attention and an increased focus on personalizing questions for symptom mapping.

“We managed to achieve a better follow-up by using individualized questions and chat rather than relying on objective measurements of weight and saturation […] We removed questions that were perceived as unnecessary.”

HCP 5 and 6_focus group 2

The HCPs experienced good internal cooperation in the RHC service team and strived for openness with each other concerning the challenges, skepticism, and lack of competence. However, a counselling service, in which all the HCPs in the RHC service team could communicate and receive guidance from a person with broad expertise in cancer and palliative care, was established to account for the lack of expertise. This guidance was perceived as pivotal by HCPs and represented a significant facilitator of the Adoption, Implementation, and Maintenance of RHC as a satisfactory follow-up for patients in the palliative phase of cancer.

## Discussion

This study reported on the use of the RE-AIM framework to assess the implementation of RHC, a technology-mediated service for home-living patients in the palliative phase of cancer. Furthermore, it explored areas of particular importance determining the sustainability of technologies for remote palliative home-based care.

Our results demonstrated that initially, HCPs felt responsible to not imposed unnecessary stress on patients, which resulted in gatekeeping behavior influencing the introduction of RHC Reach. RHC demonstrated Effectiveness by providing patients with an overview of symptom development. However, patients missed opportunities for face-to-face communication when their condition and symptoms changed. HCPs were skeptical about RHC abilities to provide palliative care and struggled to balance high-touch with high-tech care, while patients experienced poor integration and increased service complexity, which both hindered Adoption. A major issue concerning RHC Implementation was a lack of competence in HCP palliative care. For Maintenance, tailoring RHC and developing HCP-competence by providing expert advice and counselling in cancer and palliative care was applied.

### Gatekeeping

Our results suggest that HCPs experienced with cancer and palliative care distrusted the RHC potential for providing palliative care without having physical proximity to the patients and felt a great responsibility to not impose unnecessary stress and burden on patients who they perceived as very frail. These HCPs were troubled with the idea of abandoning the high-touch aspects of palliative care, which greatly affected both RHC Reach and Adoption. These results can be referred to as “gatekeeping” behavior [31, 32], which prevented RHC access to eligible patients. A review found that the fear of burdening patients was the most frequent reason for gatekeeping behavior [31]. HCPs may be hesitant and concerned that welfare technology in home-based palliative care can have a negative effect on contact with patients and result in an increased focus on the patient physical problems [33, 34], leaving the patient psychosocial, spiritual, and existential needs unattended. A prevailing opinion is that clinical care is either high-tech or high-touch, each considered antithetical to the other [7]. However, some reported research highlights benefits of using technology in palliative care, such as enhancing care accessibility and effectiveness, supporting patients to stay at home, and fostering lasting patient-HCP relationships [12, 13], which indicates that high-tech (RHC) does not necessarily excludes high-touch (person centeredness) in palliative care. Technology cannot replace personal interactions [35, 36]; however, combining remote and in-person care may be preferable to patients in the palliative phase [13].

Our results concerning HCPs’ initial skepticism of introducing RHC in home-based palliative care emphasizes the requirements to raise awareness about the benefits of integrating technology in home-based palliative care and alter negative attitudes towards combining palliative care and technology. Furthermore, patients in the palliative phase may be interested in and willing to engage in new interventions [12, 37], despite the HCP concerns. Our results suggest that once referred to RHC, patients had positive expectations regarding RHC and found it meaningful to contribute to the development of a new service that could potentially benefit others.

### Person-centered care as a key for symptom management and quality of life

RHC supports patients by offering an overview of their symptom developments, leading to improved routines in daily life. This may be significant for patients in the palliative phase, as symptom management is a prerequisite for maintaining a patient’s sense of self and improving overall well-being, quality of life, and participation in daily activities [2]. Furthermore, in line with a previous scoping review [38], our results implied that RHC provides patients with feelings of safety at home, knowing that someone is paying attention to their needs. However, as their illnesses progressed and symptoms changed, patients considered RHC a static service with limited functionality to attend to their actual needs, and they missed opportunities to communicate these needs in face-to-face meetings. These results are contradictory to a holistic, person-centered palliative care approach that considers the individual’s needs and preferences as the foundation of care [39]. This indicated a barrier to RHC Effectiveness and a key to RHC Maintenance for patients in the palliative phase. To reduce this barrier of RHC Effectiveness, measures to ensure a person-centered approach, such as the facilitation of continuous dialogue that allows patients to express what could contribute to meaning, dignity, relief, and confirmation of beliefs and values during the palliative phase, need to be considered [13, 39].

The 6 S-dialogue tool assesses patient needs in key areas of person-centered care, including self-image, symptom relief, social relationships, self-determination, synthesis and choices of strategy concerning existential and **S**piritual needs [39, 40]. These six key areas align with the World Health Organization’s guidance on palliative care as a holistic approach that addresses the emotional, spiritual, and practical needs of both patients and their families [1]. The 6 S-dialogue tool could be used by the RHC service team to facilitate continuous dialogue and assessment of patient needs, which may contribute to improving RHC Effectiveness and facilitate RHC Maintenance regarding person-centered palliative care.

### Integrating RHC in palliative care

Palliative care is a complex practice that requires a wide range of competencies from those practicing it [41], and HCP remote assessments of patient-reported symptoms depend on their knowledge and experience with the individual patient [34]. Although most HCPs had limited experience and training in cancer and palliative care, no competence-raising measures were applied prior to RHC implementation for patients in the palliative phase of cancer. The difficulties HCPs faced in assessing information transmitted by patients and uncertainties about how to act when the patients’ conditions worsened influenced the implementation of RHC. In addition, lack of knowledge influenced the Reach (gatekeeping behavior) and Adoption (distrust in RHCs potential for providing palliative care) dimensions. These results emphasize the importance of offering an educational component that ensures adequate palliative care competence in HCPs before RHC is implemented in home-based palliative care [4, 34].

Patients who live at home with a severe illness, such as cancer in the palliative phase, frequently need healthcare from different professionals and across care settings [42], which requires integrated care that is streamlined and easily navigable to facilitate access to care [43]. However, healthcare is commonly organized into silos of primary, secondary, and tertiary care levels, which may cause patients to experience great difficulty in navigating within and between each of these silos [2]. Although RHC was introduced to patients with the intention of providing an assembling service, our results suggest that for some patients, RHC became an additional silo, contributing to increased service complexity, poor integrated care, and uncertainty concerning responsibility allocations of those involved in their healthcare. This became a barrier to RHC integration in home-based palliative care. These results are closely connected to HCP experiences of struggles gaining access to relevant patient information necessary for remote palliative care, which was a challenge for Implementing RHC. Access to relevant, accurate, and timely information is a prerequisite for providing high-quality and safe healthcare [44]. RHC has the potential to improve integrated care for patients in the palliative phase of cancer; however, the necessary digital infrastructure is still lacking. There is an urgent need to establish clear lines of responsibility and a digital infrastructure that can facilitate welfare technology in home-based palliative care, such as RHC.

### Limitations

A limitation of the study may be the retrospective application of the RE-AIM framework, rather than using it to plan and guide a RHC implementation. Our retrospective application served analysis purposes and questions related to each specific RE-AIM dimension were not included in the interviews. Despite the limitations in comprehensiveness and lack of direct RE-AIM questions, the retrospective application of RE-AIM helped uncover knowledge gaps in RHC implementation. Therefore, retrospectively applying the RE-AIM framework might have revealed insights not immediately evident during initial implementation, thereby offering valuable perspectives on what was and was not successful.

Two patients participated in interviews only at baseline and 4 weeks, and one only participated at baseline. Two of these patients did not continue participation in the study because of acute health changes. We do not have information why one patient withdrew from the study after the baseline interview. This could be a limitation since their experiences regarding the use of RHC could have changed with continued use. Furthermore, we were not able to recruit family members who could have provided other perspectives and nuances regarding the RHC. The extent and access to healthcare facilities in Norway are not uniform; therefore, the outcomes and data generated from RHC implementation in rural regions may vary. This may limit the transferability of the results to other settings.

Another limitation could be that the gatekeeping behavior of the HCPs responsible for recruiting patients to the RHC might have resulted in a smaller sample size than originally planned. However, Lindgren and Lundman [45] claim that data richness may not increase with the number of participants. Furthermore, all patient and HCP participants had experience with palliative care via RHC and their willingness to share diverse experiences contributed to the depth and variety of data and generated sufficient information power [30].

The data for our secondary analysis was collected from 2017 to 2019; however a shift in welfare technology occurred during the coronavirus disease 2109 pandemic. There may have been changes in experiences regarding the provision of palliative care via RHC; thus, the strength of this study lies in capturing data under “normal” conditions, offering initial insights into the challenges and opportunities for RHC in typical circumstances.

## Conclusions

Our results suggest that the HCP gatekeeping behavior, important concerns about abandoning palliative care as a holistic and high-touch specialty, and lack of competence in palliative care affected the RHC implementation as a service to patients in the palliative phase of cancer. Although RHC facilitated improved routines in patients’ daily lives, patients experienced it a static service unable to keep up as the disease progressed, thus highlighting the need for a person-centered approach that prioritizes individual needs and preferences as the basis for providing care. Technologies, such as RHC, are not a panacea but rather an aid that can be integrated as support for an increasingly strained health service. Emphasis must be placed on establishing a digital infrastructure, with accompanying knowledge and expertise that supports this integration and sustainability of welfare technologies, such as RHC. The results of this study could be of importance to others implementing technologies for remote care of patients in a palliative phase or those with complex care requirements regardless of disease.

The RE-AIM dimensions provide significant insight to understand implementation and sustainability of welfare technologies, such as RHC, in future healthcare. In future studies of welfare technology implementation for home-based palliative care, we recommend employing the RE-AIM framework as a guiding tool for planning and executing the implementation process.

Considering the complex, individual care needs and patient vulnerability in home-based palliative care, future research should prioritize exploring professional and ethical considerations surrounding welfare technologies implementation because these technologies could potentially add unintended burdens to this already vulnerable group. Thus, it is crucial to understand the professional considerations and ethical implications, as they might pose challenges related to gatekeeping behaviors that may challenge RHC implementation and potentially hinder patient access to care.

## Data Availability

Data are available upon reasonable request from the corresponding author.

## Abbreviations

COPD: chronic obstructive pulmonary disease
HCP: healthcare professional
RE-AIM: Reach Effectiveness Adoption Implementation Maintenance
RHC: remote home care
